# Supplementary material for: Preconception mental health (Healthy Life Trajectories Initiative): Identifying factors associated with probable anxiety and depression among young women living in urban-poor South Africa
Source: PLOS Ment Health. 2026 Mar 24;3(3):e0000578. doi: 10.1371/journal.pmen.0000578 (PMC13012478; doi:10.1371/journal.pmen.0000578)
Supplement: S1 Table — (DOCX) [file pmen.0000578.s001.docx]

**S1 Table. Generalised structural equation model in a sample of respondents for socioeconomic status, marital status, sleep and mental health** (*n*=7377)

| **Exposure** | **Outcome**  (*n*=6467) | **Direct effects (CI)** | ***p* value** | **Indirect effects (CI)** | ***p* value** | **Total effects (CI)** | ***p* value** | **% mediated** |
| --- | --- | --- | --- | --- | --- | --- | --- | --- |
| **Model 3** | | | | | | | | |
| **Poverty on poor sleep via depression (binary)** | | | | | | | | |
| Poverty | PHQ9 >10 (probable) | 0.044 (-0.001; 0.089) | 0.058 | 0.031 (-0.002; 0.064) | 0.067 | 0.068 (0.026; 0.112) | **0.002** | - |
| **Poverty on poor sleep via anxiety (binary)** | | | | | | | | |
| Poverty | GAD7 >10 (probable) | 0.057 (0.017; 0.98) | **0.005** | 0.050 (0.013; 0.088) | **0.009** | 0.088 (0.042; 0.133) | **<0.001** | 56.8%† |
