# Supplementary figures and images for: Preconception mental health (Healthy Life Trajectories Initiative): Identifying factors associated with probable anxiety and depression among young women living in urban-poor South Africa
Source: PLOS Ment Health. 2026 Mar 24;3(3):e0000578. doi: 10.1371/journal.pmen.0000578 (PMC13012478; doi:10.1371/journal.pmen.0000578)

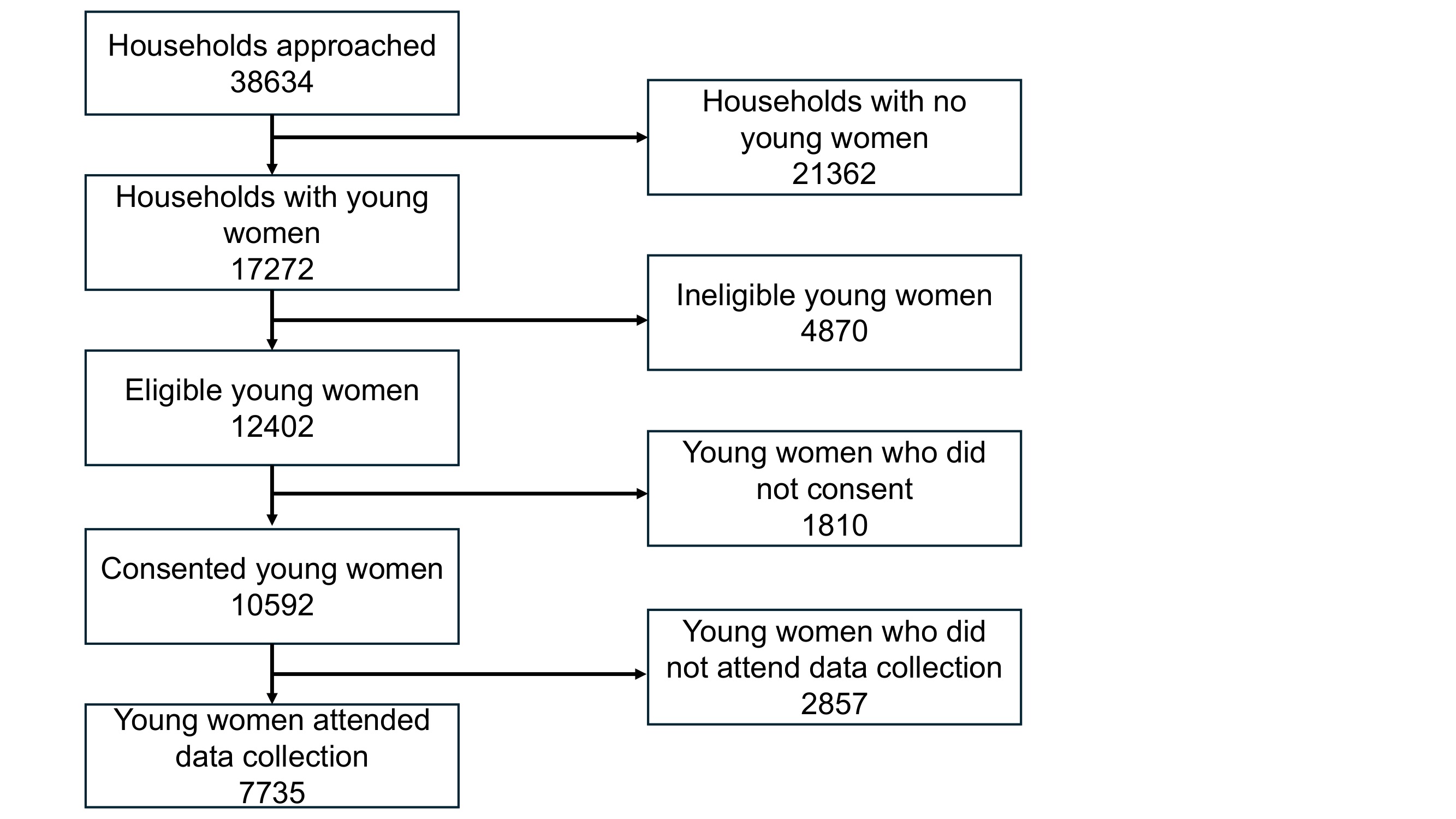

Supplement: S1 Fig — (TIFF) [file pmen.0000578.s002.tiff]
