# Supplementary material for: Preconception mental health (Healthy Life Trajectories Initiative): Identifying factors associated with probable anxiety and depression among young women living in urban-poor South Africa
Source: PLOS Ment Health. 2026 Mar 24;3(3):e0000578. doi: 10.1371/journal.pmen.0000578 (PMC13012478; doi:10.1371/journal.pmen.0000578)
Supplement: Attachment — Submitted filename: HeLTI MH Rebuttal_PLOS MH.pdf [file pmen.0000578.s004.pdf]

DPHRU
MRC/Wits Developmental
Pathways for Health Research Unit

16/10/2025

Editor-in-Chief
Professor Rochelle Burgess
University College London
UK

Dear Professor Burgress,

Resubmission of Revised Manuscript PMEN-D-25-00305

Thank you for the opportunity to resubmit our original research article, “Preconception mental health (Healthy Life Trajectories Initiative): Identifying factors associated with probable anxiety and depression among young women living in urban-poor South Africa,” for consideration in PLOS Mental Health.

We carefully addressed all reviewer and editorial comments.

Enclosed are:

- A point-by-point rebuttal, with each comment and our response.
- A clean revised manuscript and a tracked-changes version highlighting all amendments.

In response to the reviewers’ guidance, we have strengthened the rationale and methods, clarified analytic decisions, refined the presentation of results, and expanded the discussion of implications for policy and practice. We believe these revisions have materially improved the clarity, rigor, and contribution of the paper.

We hope the revised manuscript meets the journal’s expectations and would be pleased to respond to any further queries.

Yours sincerely,

Prof Shane Norris
Corresponding author, and on behalf of all co-authors

Reviewer #1

1. Statistical analysis of the results obtained and the coding method were extensively tabulated. However, the study's purpose remains somewhat unclear without suggesting a definite path towards addressing apartheid influenced systemic barriers, which contribute to the increase in anxiety and depression for the socioeconomically disadvantaged young women population in South Africa. Since the study conclusion is substantiated with evidence that early life trauma is a precursor to the increased risk for anxiety and depression among young women in urban poor settings, a glaring study limitation still exists because the study results only loosely mentioned the need for mental health, social support for the vulnerable population. It fails to identify concrete steps for addressing structural inequalities and social injustices, as these unfortunately play a significant role in creating the environment upon which anxiety/depression breeds in later years in the lives of young women living in Urban poor South Africa.

   Response:

   Thank you for highlighting the need to articulate a clearer pathway from our findings to structural action and limitations. We have: (i) strengthened the Introduction; (ii) expanded the limitations section to note that we did not directly measure several structural exposures and propose future work; and (iii) expanded the conclusion with a pragmatic evaluation agenda combining mental-health care with social-protection and employment interventions.

Reviewer #2

1. The conclusions, particularly those derived from the generalized structural equation modelling (gSEM), are currently overstated. For instance, the interpretation of the generalized structural equation model (gSEM) presents a simplified, unidirectional view of what are likely complex, bidirectional relationships. The modelled path from poor sleep to depression is a prime example. The clinical and empirical literature strongly supports a reciprocal feedback loop where depression causes poor sleep and poor sleep exacerbates depression. The current model and its conclusions only reflect one direction of this effect. Therefore, a conclusion that the effect of poverty on depression is "fully mediated by sleep" is an oversimplification not fully supported by the data, as it does not account for this inherent bidirectionality.

   Response: We appreciate the reviewer's insightful comment regarding the bidirectional nature of the relationship between sleep and depression. We agree that existing literature supports a reciprocal feedback loop between these two variables. In our analysis, we initially hypothesised and tested a bidirectional association between sleep and depression within the gSEM model. However, in this cohort, the reverse path (from depression to sleep) was not statistically significant. As a result, we retained the unidirectional path (from poor sleep to depression) for parsimony and model fit. We have revised the manuscript to clarify this analytical decision.

   "...Bidirectional paths between sleep and depression were initially tested within the model; however, as the reverse path (from depression to sleep) was not statistically

significant, the final model retained only the unidirectional path to improve model fit and interpretability."

2. Regarding question 2, while the choice of statistical methods (logistic regression, gSEM) is appropriate for the research questions, the application and reporting lack the necessary rigor. The presentation of the logistic regression results in Table 4 as a series of separate, unadjusted models is not ideal. A hierarchical modeling approach would more clearly demonstrate the independent contribution of various factors.

   <u>*Response*</u>*:*

   Thank you for the suggestion and we agree that the a more hierarchical approach would add clarity and we have updated the methods and reworked Table 4.

   We have re-run logistic regressions as hierarchical blocks

   For each outcome:

   - *Block 0 (base socio-demographics & household): age, education (2 indicators), relationship status, parity, household SES, household size.*
   - *Block 1 (+ early adversity): add ACE count.*
   - *Block 2 (+ current deprivation): add food insecurity.*
   - *Block 3 (+ health behaviours/sleep): add PA (hrs/wk), hazardous alcohol, poor sleep.*
   - *Block 4 (+ comorbidity): add the other symptom (e.g., add moderate–severe anxiety in the depression model; omit for anxiety-as-outcome, or vice-versa per your conceptual model).*

   At each step, we report adjusted odds ratios (95% CIs), changes in model fit (overall model significance, Δpseudo-R^2), and likelihood-ratio tests comparing successive blocks

3. The theoretical justification for the a priori gSEM model is underdeveloped. The specific pathways tested need a stronger grounding in existing literature or theory.

   <u>*Response:*</u>

   Thank you for requesting clearer theory. We have expanded the methods explaining the underlying established frameworks for the gSEM (social determinants of health and the stress process) Specifically, we justify SES effects operating indirectly through food insecurity, poor sleep, unemployment, social support, and alcohol use, and directly, with ACEs modelled as upstream exogenous risks.

4. My justification for the negative response to Question 3 is direct. The manuscript does not meet the journal's data availability requirements. The authors' Data Availability Statement claims that all data are included within the article and its supplements. However, a review of these documents confirms they contain only aggregated summary

statistics. The PLOS Data Policy explicitly requires that the individual, de-identified data points underlying these summary statistics (e.g., the data points behind means, medians, and variance measures) be made fully available. To comply, the authors must deposit a de-identified participant-level dataset in an appropriate public repository and include the access information in their statement.

Response: *We thank the reviewer for highlighting this important point regarding data availability. We acknowledge that the manuscript currently reports only aggregated summary statistics and does not include individual-level data. To address this, we have clarified in the manuscript that the data codebook and data is publicly available on the HeLTI website (https://helti-hub.tghn.org/cohorts/south-africa/). We believe this approach aligns with the journal's policy for controlled access to participant-level data when open deposition is not possible due to ethical or governance considerations. This information has now been clearly reflected in the manuscript.*

5. Finally, my affirmative response to Question 4 is based on the overall quality of the writing. The manuscript is presented in an intelligible fashion and is written in standard, professional English. The narrative is logical and accessible to an expert in the field. However, to fully meet the highest standards of clarity and precision, the manuscript would benefit from a final copyedit. During the review, several important errors were noted that should be corrected:

   i. Inconsistencies: There are discrepancies between the results described in the text and the data presented in the tables. For example, the text reports a non-significant confidence interval for the association between being in a committed relationship and anxiety, whereas Table 4 reports a significant one. All such values must be checked meticulously.

      Response: *We thank the reviewer for bringing these errors to our attention. We apologise for the oversight, and the errors have now been corrected. All amendments made throughout the manuscript have been tracked in Track Changes for transparency.*

   ii. Table Accuracy: The frequencies in Table 1 for marital status and education doesn't add up when manually added the frequencies for lower SES and higher SES to the total population, i.e., "Married" status do not sum correctly (3 + 51 ≠ 83). All tables should be reviewed for accuracy.

      Response: *We thank the reviewer for highlighting this point. The apparent discrepancy arises as a result of the "Total Population" column that represents the full sample of women (n=7735), whereas the lower SES and higher SES columns reflect only participants who completed the household SES questionnaire (n=7388). As a result, the sum of lower and higher SES does not equal the total population. This stratification was limited to respondents with valid SES data, which explains why the subgroup totals differ from the overall population counts. We have clarified this in the table legend and reviewed all tables to ensure consistency.*
