# Supplementary material for: Preconception mental health (Healthy Life Trajectories Initiative): Identifying factors associated with probable anxiety and depression among young women living in urban-poor South Africa
Source: PLOS Ment Health. 2026 Mar 24;3(3):e0000578. doi: 10.1371/journal.pmen.0000578 (PMC13012478; doi:10.1371/journal.pmen.0000578)
Supplement: Attachment — Submitted filename: HeLTI MH Rebuttal_PLOS MH_R2.pdf [file pmen.0000578.s005.pdf]

27/01/2026

Editor-in-Chief
Professor Rochelle Burgess
University College London
UK

Dear Professor Burgress,

Resubmission of Revised Manuscript PMEN-D-25-00305R1

Thank you for the opportunity to resubmit our original research article, “Preconception mental health (Healthy Life Trajectories Initiative): Identifying factors associated with probable anxiety and depression among young women living in urban-poor South Africa,” for consideration in PLOS Mental Health.

We carefully addressed all reviewer and editorial comments.

Enclosed are:

- A point-by-point rebuttal, with each comment and our response.
- A clean revised manuscript and a tracked-changes version highlighting all amendments.

In response to the reviewers’ guidance, we have strengthened the rationale and methods, clarified analytic decisions, refined the presentation of results, and expanded the discussion of implications for policy and practice. We believe these revisions have materially improved the clarity, rigor, and contribution of the paper.

We hope the revised manuscript meets the journal’s expectations and would be pleased to respond to any further queries.

Yours sincerely,

Prof Shane Norris
Corresponding author, and on behalf of all co-authors

Reviewer Comments to the Author

REVIEWER 2

PLOS Mental Health's publication criteria

1. Results text misreports the depression OR for hazardous alcohol as 3.18 (which matches the poor sleep OR) (Manuscript PDF p.15/39 vs Table 4 Model 4, Manuscript PDF p.17/39).

   Response: We thank the reviewer for bringing this error to our attention. The typo has been corrected in the revised manuscript using track changes.

2. The discussion states food insecurity was "significantly associated with poorer mental health outcomes" (Manuscript PDF p.24/39), but adjusted logistic regressions show non-significant associations for food insecurity across outcomes (Table 4 Models 3–5; Table 5) (Manuscript PDF pp.17–18/39).

   Response: Thank you for pointing this out. We acknowledge this oversight in the reported findings and have corrected the discussion accordingly in the revised manuscript.

3. Consuming a hazardous amount of alcohol... depression: OR 3.18 is mentioned in narrative. But, in Table 4, (Model 4), Alcohol OR is 1.29

   Response: Thank you for highlighting this. We acknowledge this and have amended the manuscript accordingly.

4. Table 4 (Anxiety Model 4) and Table 5 report identical coefficients (e.g., Sleep OR 4.03), The Given the different outcomes and sample size, this looks surprising. I would like to request the authors to recheck this.

   Response: Thank you for highlighting this valid point. We have carefully rechecked the models reported in Table 4 (Anxiety Model 4) and Table 5. Odds ratios and 95% confidence intervals were rounded to two decimal places for presentation thus, the estimates for some variables coincide by chance across the two models. We can confirm that this reflects rounding for reporting purposes rather than a duplication or modelling error.

5. Table 4 reports hazardous alcohol as highly significant for anxiety (1.09*), while the CI spans 1.00 (0.85;1.39) (Manuscript PDF p.17/39).

   Response: Thank you for pointing this out. We acknowledge this oversight in the reported findings and have corrected the discussion accordingly in the revised manuscript.

6. Table 6 contains implausible CI values (e.g., -139 to 139; -164 to 164) for poverty→(depression/anxiety) via food insecurity (Manuscript PDF p.21/39), as well as formatting errors in at least one CI cell (Manuscript PDF p.21/39).

   Response: Thank you for highlighting this issue. Following your comment, we re-evaluated our model and revised the food insecurity variable from a binary to a multinomial specification. As a result, the model was rerun, and new coefficients and confidence intervals are now presented in the revised manuscript. This adjustment substantially improved model fit (AIC: 72603.36; BIC: 72914.14) and resulted in plausible confidence intervals for all pathways, including the pathway from poverty to depression/anxiety via food insecurity. Additionally, we also amended Figure 1 to present all pathways to explicitly assess

bidirectional pathways between depression as the outcome (Model 1) and anxiety as the outcome (Model 2), as shown in the revised Figure 1.

Statistical analysis

7. While data missingness is acknowledged and variable n's appear in descriptive tables (Manuscript PDF p.13/39), the manuscript does not state how missing data were handled in logistic regression and gSEM (complete-case vs imputation), nor does it provide analytic sample sizes for each model.

   Response: We thank the reviewer for this important comment. We have now added a clear description of how missing data was handled in the statistical analyses. Specifically, all models were run using complete-case analysis, with participants missing data on any of the variables included in a given model excluded from that model. Additionally, we have included the analytic sample sizes for each model in the respective Tables and clarified this in the statistical analysis section of the revised manuscript.

   "…All regression models (logistic and gSEM) were conducted using complete-case analysis, whereby participants with missing data on any variables included in each model were excluded from that analysis."

8. Methods specify RMSEA and BIC as fit criteria (Manuscript PDF p.13/39), but realised RMSEA/BIC are not reported. Given the centrality of gSEM in the conclusions, fit statistics should be presented.

   Response: Thank you for this comment. For the gSEM analyses, model fit was assessed using information criteria (AIC and BIC), which are appropriate for gSEM frameworks. We have now added the AIC and BIC values for all gSEM models to the revised manuscript as requested.

9. Methods state sensitivity analyses across SES strata were conducted (Manuscript PDF p.13/39), but no results are presented (Not stated/unclear). Please report these.

   Response: We have corrected this in the methods section.

   "We performed a stratification analyses to examine SES strata differences for sociodemograhic and outcome variables"

REVIEWER 3

1. The Odds Ratio of 32.20 (95% Confidence Interval: 24.34–42.60) shows a very high link between moderate-to-severe anxiety and depression. This suggests that, in this study, anxiety and depression may be viewed as almost the same thing. The GAD-7 and PHQ-9 questionnaires share common symptoms, such as sleep problems and restlessness. The authors should clarify whether these tools are differentiating between two different conditions or just measuring general psychological distress.
2. Check for overlap between GAD-7 and PHQ-9 scores. Perform a Confirmatory Factor Analysis (CFA) to show that these scores represent different factors in this sample. Consider whether symptom overlap (like sleep items included in both questionnaires) inflates this odds ratio. It may be useful to run the analysis without the "sleep" item in either score to see if the odds ratio changes. Revisit Model 5. Using one mental health condition as a predictor for another in logistic regression may obscure the effects of variables like SES and ACEs due to excessive modification.

Response: Thank you for this insightful comment. We acknowledge that some items within these instruments overlap (e.g., sleep disturbance and restlessness), which may contribute to the strong observed association between moderate-to-severe anxiety and depression. In response to this concern and as suggested by the reviewer, we conducted a confirmatory factor analysis (CFA) to assess whether the scales capture distinct latent constructs rather than general psychological distress. A two-factor CFA was specified, with a latent depression factor indicated by the nine PHQ9 items and a latent anxiety factor indicated by the seven GAD7 items, allowing the two latent variables to covary. Model fit indices indicated good fit to the data ($\chi^2(103)=2392.16$; RMSEA=0.054; CFI=0.943; TLI=0.934; SRMR=0.030; CD=0.925), supporting the distinction between anxiety and depression constructs despite their expected correlation. Given the conceptual overlap between psychomotor agitation/restlessness (PHQ9 item 8; GAD7 item 5) and physiological arousal (PHQ9 item 4; GAD7 item 4), we conducted sensitivity analysis firstly removing PHQ9 item 8 from the measurement model. The resulting model demonstrated near-identical fit ($\chi^2(89)=2210.50$; RMSEA=0.056; CFI=0.945; TLI=0.935; SRMR=0.030; CD=0.924), and then another model removing PHQ9 item 4 (($\chi^2(89)=2078.86$; RMSEA=0.052; CFI=0.947; TLI=0.937; SRMR=0.030; CD=0.919), indicating that symptom overlap did not materially influence the factor structure or inflate associations between anxiety and depression in this sample.

With respect to Model 5, we agree that adjusting for one mental health condition when modelling the other may attenuate the effects of upstream determinants such as SES and ACEs. Sensitivity analyses removing the overlapping item from the GAD7 attenuated the odds ratio for anxiety predicting depression from OR 32.2 to OR 26.9, but the association remained highly significant (95% CI 20.1–36.0; $p<0.001$), indicating that the strong association was not fully attributable to symptom overlap. We have also clarified this in the revised manuscript.

[1] Hart C, Draper CE, Soepnel LM, Godongwana M, Mabetha K, Nyati LH, et al. Examining the psychometric properties of the Patient Health Questionnaire-9 and Generalized Anxiety Disorder-7 among young urban South African women. J Affect Disord. 2025;369:61–70.

"… To assess potential overlap between anxiety and depression symptoms, we conducted a confirmatory factor analysis (CFA) with latent variables for PHQ9 and GAD7..."

"… CFA supported distinct latent factors for anxiety and depression. In model 5, removal of the overlapping GAD7 item attenuated the OR for anxiety predicting depression, however, the association remained highly significant (OR 26.9 [95% CI 20.1; 36.0) (results not shown), indicating that the strong association was not fully attributable to symptom overlap."

3. The manuscript often uses words like "drive a syndemic," "influence," and "effects." Since the data is collected at a single point in time, we cannot determine the direction of the relationship (for example, whether Depression leads to Sleep issues or sleep issues lead to Depression). We can only make theoretical assumptions. The authors need to provide stronger statistical support for rejecting the idea that sleep and depression affect each other.
4. Recognize the limits of generalized Structural Equation Modeling (gSEM) with this cross-sectional data. Replace terms like "effects" and "influences" with "associations" or "pathways" throughout the Results and Discussion sections. Clarify why the authors rejected the "Depression leads to Sleep" path in favor of "Sleep leads to Depression." Did they base this on model fit alone (using the Bayesian Information Criterion)? They need to explain their reasoning, as sleep problems are a key symptom of depression, suggesting that the causal relationship may work in both directions

Response: Thank you for this important clarification. We agree that, given the cross-sectional nature of the data, causal directionality between sleep and depression cannot be definitively established, and any directional pathways tested in the gSEM are theoretically informed rather

than causal. While we initially evaluated a bidirectional pathway between sleep and depression, the reverse path (i.e., depression to sleep) was not statistically supported in this dataset: the path coefficient was small and non-significant, and including this path did not improve overall model fit based on the Information Criteria used to assess gSEM model fit. For transparency, however, we have included the results of this analysis as a table in the Supplementary Material of the revised manuscript (Supplementary Table S1). However, we acknowledge that the absence of statistical significance does not constitute strong evidence against bidirectionality, particularly in cross-sectional analyses.

In response to this comment, we have also revised the manuscript to (i) temper causal language (e.g., replacing terms such as “drive,” “influence,” and “effects” with “associations” or “pathways”), and (ii) explicitly state that the gSEM reflects a theoretically guided model rather than a causal framework. We have also expanded the discussion to acknowledge the well-established reciprocal relationship between sleep and depression in the literature and to clarify that alternative bidirectional explanations remain plausible and cannot be ruled out with the current study design.

5. The study uses a household asset score. While this is a common approach, it may not effectively differentiate between the "poorest of the poor" and the "relatively stable poor" in a similar urban setting. This could limit the observed effects related to social structure.

   Response: Thank you for this comment. We agree that while household asset score is widely used to capture SES in LMIC urban settings, they may not fully distinguish between the heterogeneity within a urban context. We agree with the reviewer that this limitation may attenuate observed associations related to social structure. We have now explicitly acknowledged this limitation in the revised manuscript.

   “…Socioeconomic status was assessed using a household asset score, which, although commonly applied, may not fully differentiate between the heterogeneity within a similar urban context, potentially attenuating observed associations related to social structure.”

6. In the Discussion section, likely in the Limitations part about self-reporting. It is suggested to refer from the paper: “Mental Health Monitoring and Intervention Using Unsupervised Deep Learning on EEG Data “. The manuscript notes that self-reported mental health symptoms can be influenced by social desirability bias. This reference is important because it contrasts traditional survey methods with new objective approaches. It strengthens the manuscript by highlighting that, while self-reports are useful (as seen in this study), future mental health methods may rely more on objective measures (such as EEG) and unsupervised learning to minimize misreporting and labeling issues. This places the study's methodology within the changing landscape of mental health diagnostics. The following reference can improve the manuscript’s discussion on limitations, especially regarding self-reported versus objective measures, the pandemic context of the data, and future technological implications of their proposed interventions.

   Response: Thank you for this suggestion. We agree that self-reported mental health measures, while validated and appropriate for the current study, are subject to reporting bias. We have now expanded the limitations section of our revised manuscript to reference emerging objective approaches to mental health assessment, including the suggestion the reviewer made.

   “… Lastly, women self-reported symptoms in a face-to-face interview, which may have introduced social desirability bias. While appropriate for population studies, future approaches using objective measures may help reduce misreporting and improve mental health assessment accuracy, as highlighted in recent work on EEG-based monitoring methods.”
